# Cervical Cancer Associated with Pregnancy: Current Challenges and Future Strategies

**DOI:** 10.3390/cancers16071341

**Published:** 2024-03-29

**Authors:** Jennifer Le Guévelou, Lise Selleret, Enora Laas, Fabrice Lecuru, Manon Kissel

**Affiliations:** 1 Faculty of Medicine, University of Geneva, 1205 Geneva, Switzerland; 2 Department of Radiation Oncology, Centre Eugène Marquis, 35000 Rennes, France; 3 Department of Gynaecology and Obstetrics, Tenon University Hospital, Assistance Publique des Hôpitaux de Paris (AP-HP), Sorbonne University, 75005 Paris, France; 4 Cancer Associé à La Grossesse (CALG), French CALG Network, 75005 Paris, France; 5 Breast, Gynecology and Reconstructive Surgery Unit, Institut Curie, 75005 Paris, France; 6 Université de Paris, 75006 Paris, France; 7 Department of Radiation Oncology, Institut Curie, 75005 Paris, France

**Keywords:** cervical cancer, pregnancy, chemotherapy, radiation therapy, fetal outcome

## Abstract

**Simple Summary:**

Cervical cancer occurring during pregnancy represents a rare and challenging situation and requires multidisciplinary care. Both diagnosis and treatment should take into consideration maternal and fetal health, while not compromising the chances for cure. Currently, guidelines for the management of cervical cancer occurring during pregnancy are provided by international consensus meetings with expert panels. However, the current guidelines do not discuss the various approaches that can be found within the literature, such as alternative staging modalities or innovative surgical approaches. Our systematic review, performed with the “Cancer Associé à La Grossesse” (CALG) network, aims to fill the gap on current issues, including both neoadjuvant chemotherapy and surgical strategies to preserve fertility.

**Abstract:**

Cancer during pregnancy is defined as a tumor diagnosed in a pregnant woman or up to 1-year post-partum. While being a rare disease, cervical cancer is probably one of the most challenging medical conditions, with the dual stake of treating the cancer without compromising its chances for cure, while preserving the pregnancy and the health of the fetus and child. To date, guidelines for gynecological cancers are provided through international consensus meetings with expert panels, giving insights on both diagnosis, treatment, and obstetrical care. However, these expert guidelines do not discuss the various approaches than can be found within the literature, such as alternative staging modalities or innovative surgical approaches. Also, the obstetrical care of women diagnosed with cervical cancer during pregnancy requires specific considerations that are not provided within our current standard of care. This systematic review aims to fill the gap on current issues with regards to the management of cervical cancer during pregnancy and provide future directions within this evolving landscape.

## 1. Introduction

Cancer during pregnancy is defined as a tumor diagnosed in a pregnant woman or up to 1-year post-partum. Among them, cervical cancer is one of the most frequently diagnosed, alongside breast and ovarian cancers [1,2,3], and occurs in approximately 1.6–11.1 cases per 100,000 pregnancies, with 3% of cervical cancers being diagnosed during pregnancy [4,5,6,7]. Its incidence is on the rise, with a crude increase of 2.9% suggested over a 30-year period in the Danish Cancer Registry [8].

Cervical cancer during pregnancy is probably one of the most challenging medical conditions, with the dual stake of treating the cancer without compromising its chances for cure, while preserving the pregnancy and the health of the fetus and child. Over the years, the treatment strategy has gradually changed from radical treatment with termination of pregnancy to more conservative approaches, allowing even fertility-sparing strategies [9,10,11,12]. To date, guidelines for gynecological cancers have been provided through international consensus meetings with expert panels [10,11,12], giving insights on both diagnosis, treatment, and obstetrical care. Since the publication of the 3rd edition of these guidelines in 2019, several articles have reported advances in both diagnosis and treatment that may improve the management of these patients.

This systematic review aims to fill the gap on current issues with regards to the management of cervical cancer during pregnancy and provide future directions within this evolving landscape.

## 2. Materials and Methods

### 2.1. Eligibility Criteria

All studies reporting data on the management of cervical cancer during pregnancy were included in this systematic review. Studies were deemed eligible if they reported either oncological, obstetrical, or pediatric outcomes.

### 2.2. Information Sources and Search Strategy

A systematic review was performed on PubMed in January 2022 and updated in August 2023. We used the Preferred Reporting Items for Systematic Reviews and Meta-analysis (PRISMA) guidelines for reporting [13]. The keyword “pregnancy” (MeSH term) and “cervix cancer” (MeSH term) were used. The systematic review followed the PRISMA recommendations. The protocol has not been registered.

### 2.3. Selection Process

A consensus on article selection was found between two reviewers (J.L.G. and M.K.). For every study, the following data were retrieved: publication year, number of patients, study design, tumor histology, stage, term of pregnancy, treatment, delivery modality, and maternal and fetal outcomes. Pediatric outcomes were also retrieved, if available.

### 2.4. Synthesis Method

All studies meeting the inclusion criteria were selected for narrative synthesis. The results have been reported narratively and summarized in tables when appropriate.

## 3. Results

**Figure 1:**
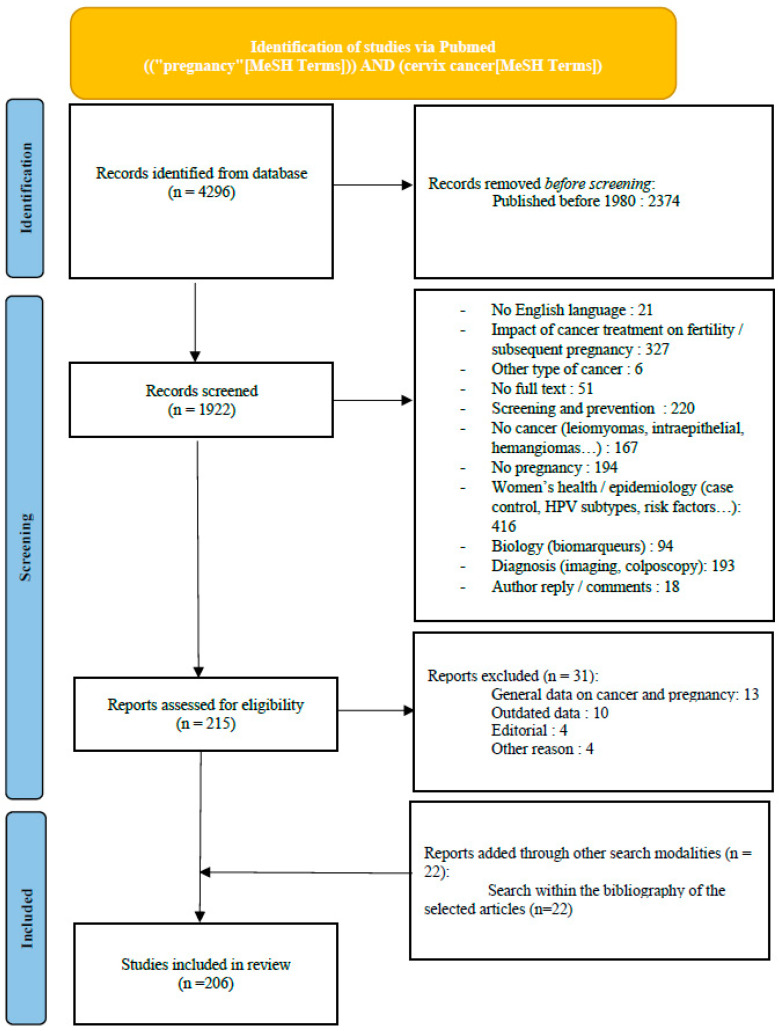
Flow chart.

A flow chart of the literature screening is shown in Figure 1. The search allowed retrieval of a total of 4296 articles. The research was narrowed to articles published after 1980 in order to exclude outdated data. A total of 1922 articles were screened. After reading both titles and abstracts, 1707 articles were excluded (diagnosis technique, impact of cancer treatment on fertility, prevention…). Two hundred and fifteen articles were assessed for eligibility. Thirty-two articles were further excluded. We added 22 articles through searches within the bibliographies of other articles. Finally, 206 articles were included in this review.

A total of 67 cases and 30 reviews were collected and reported in Supplementary Tables S1 and S2, respectively.

### Cervical Cancer during Pregnancy: Opportunity for Screening or Risk-Factor?

Approximately 30% of women diagnosed with cervical cancer are in their childbearing years, with a maximum of incidence reached around the age of 40 [14]. Taken together with the tendency to postpone childbirth in developed countries, these facts might explain the rise in incidence in cervical cancer diagnosed during pregnancy in the past decades [8].

Cervical cancer is one of the most frequently diagnosed cancers during pregnancy, alongside breast and ovarian cancers [1,2,3,8]. The incidence of cervical cancer is lower in pregnant women than in non-pregnant women. However, the incidence of cervical cancer during pregnancy is on the rise, which may partly be explained by the current tendency to delay childbearing. Data obtained from large cancer networks indicate that pregnant patients are more likely to be diagnosed with early-stage cervical cancer, with only 26% of them diagnosed with stage II-IV cancer, compared to 52% in non-pregnant women [15]. Yet, data from national registries remain quite rare and this finding may not be applicable to other countries. Whether pregnancy is a risk factor for development of cervical cancer or not remains an unanswered question. Persistent human papilloma virus (HPV) is firmly established as the main carcinological mechanism associated with the development of both cervical squamous cell carcinoma (SCC) [16] and adenocarcinoma [17]. Estradiol, for which blood levels are elevated during pregnancy, has been suggested to be associated with development of HPV-related tumors, through both inactivation of retinoblastoma protein (pRb) and downregulation of phosphatase and tensin homolog (PTEN) [18]. Additionally, estrogens have long been suggested to influence the development of cervical adenocarcinomas [19,20]. Also, immunohistochemical analyses of cervical cancers diagnosed during pregnancy revealed that almost all tumors express estrogen receptors [21]. As high-level evidence is lacking, a hormone-dependent carcinogenesis cannot be entirely excluded. Last but not least, the shift in maternal immunity, with both inhibition of the Th1 pathway and enhancement of the Treg pathway, has been shown to enhance immune tolerance, which could ultimately lead to cervical cancer progression [22]. Specific investigations on both tumor biology and tumor micro-environment are warranted before making any conclusions on this subject.

## 4. Diagnosis

As for non-pregnant women, cervical cytology with HPV testing, colposcopy, and biopsy can be safely performed during pregnancy. Cervical curettage, which can be performed as a complementary procedure at the time of colposcopy, should not be performed on pregnant women, as it is associated with both an increase in abortion and premature delivery rate [23].

Magnetic resonance imaging (MRI) is one of the reference imaging modalities for cervical cancer and allows assessment of both local (parametrium, vagina, uterus) and regional extension [24,25]. Due to its non-ionizing properties, it is the preferred imaging modality during pregnancy and has proven non-deleterious effects on the fetus regardless of the term of the pregnancy [26]. Current guidelines advocate against the use of Gadolinium [12], both due to its low added value in parametrial evaluation and due to its risks for the fetus [27]. However, it may provide a benefit in detecting small invasive cervical lesions. The 11th European Symposium on Urogenital Radiology recently stated in favor of the administration of Gadolinium to pregnant women, if deemed necessary [28].

The challenges associated with pelvic MRI in pregnancy are multiple. First, T2 sequences can be artifacted due to fetal movement, thereby limiting their interpretation. Second, cervical distension occurring during pregnancy can complicate the measurement of the tumor size. Only one trial assessed the clinical impact of pelvic MRI in the particular case of cervical cancer occurring during pregnancy. It showed its value in both diagnosis and follow-up under neoadjuvant chemotherapy (NAC). A good correlation between MRI findings and pathology specimens was also demonstrated [29]. Fetal movements had no consequence on MRI interpretation and could be minimized by using ultrafast MR sequences.

The pitfalls of MRI usually include its low specificity with regards to lymph node evaluation [30]. This evaluation can be even more challenging during pregnancy, because of the ectopic decidual reaction that can occur in the pelvic and para-aortic lymph nodes [31,32,33]. In this setting, dual positron emission tomography (PET)/MR imaging showed both a high sensitivity and a high specificity for local and nodal extension [34], even if the small size of the cohort precludes any conclusions on both sensitivity or specificity. If the fetal radiation dose after administration of ^18^F-FDG remains a concern, Zanotti-Fregonara et al. reported it to range from 5.2 mSv in early pregnancy to 1.4 mSv in late pregnancy [35], which remains well below the thresholds for deterministic effects (Table 1).

To date, guidelines advocate performing pelvic +/− para-aortic lymph node dissections as a staging modality both for tumors smaller or larger than 2 cm diagnosed before 22 to 25 weeks of pregnancy. PET/MRI may be of future value for patients that cannot undergo the lymph node dissection procedure (i.e., in their third trimester of pregnancy). Additional studies are necessary to determine both the benefit and safety of this procedure in pregnant patients.

### 4.1. Cancer Treatment during Pregnancy

#### 4.1.1. General Considerations

In situations where pregnancy preservation is not aimed for, termination of pregnancy should be performed without delay and cancer management should be similar to that for non-pregnant women. Both termination of pregnancy and diagnosis of cancer represent situations at high-risk of emotional distress, so the patient should be counseled towards specific psychological care.

Specific attention should be given to symptoms caused by the tumor. In the presence of major bleeding, cancer treatment should be initiated without delay, regardless of the term of the pregnancy.

#### 4.1.2. Surgery

##### Conization and Trachelectomy

Conization with clear margins is an option in international guidelines as the treatment for T1a1–T1a2 squamous carcinoma, regardless of LVSI status and T1b1 LVSI negative tumors with negative nodes [36]. However, during pregnancy, conization is tricky since it may cause bleeding (5–15%), spontaneous abortion, preterm premature rupture of membranes, preterm labor, and infection. Data are conflicting on the subject, with authors reporting up to 5–15% bleeding rates and spontaneous abortion rates of 25% and some other authors reporting no significant intraoperative or post-operative complications and deliveries at term, with/without cerclage placement [37,38,39,40,41,42,43,44,45,46,47,48,49]. Goldberg suggested a cone cerclage prior to conization to prevent major hemorrhage [46]. Cold-knife conizations have been evaluated in large but ancient series [39,40]. Modern literature reports prefer loop-excision or more marginally laser conization [38,41,44,50,51]. Loop excision may be the preferred technique but with higher rates of non-in sano resections. The ideal timing during pregnancy also remains to be determined: some authors advocate for the first trimester given the cervix is not as hyperemic at this stage, but some other authors advocate waiting until the second trimester to avoid the association of the procedure with a spontaneous, and likely unrelated, miscarriage [44,50,52,53].

**Figure 2:**
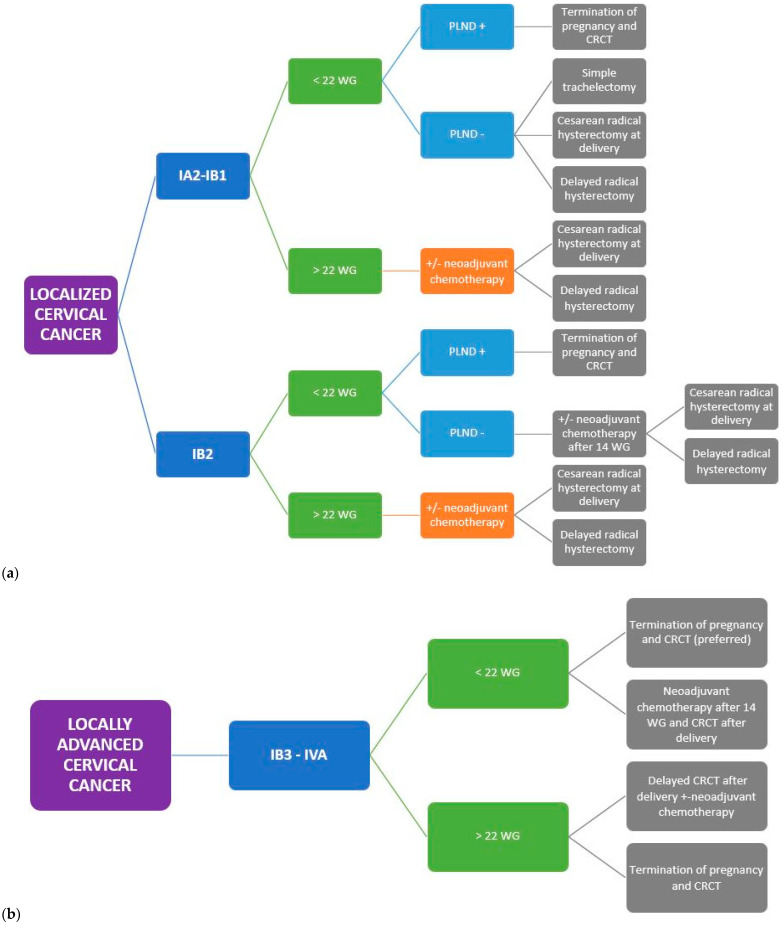
(**a**) Summary of the management of localized cervical cancer occurring during pregnancy. (**b**) Summary of the management of locally advanced cervical cancer occurring during pregnancy Abbreviations: WG: weeks of gestation, PLND: pelvic lymph node dissection, CRCT: concomitant radio-chemotherapy.

Current guidelines advocate that simple trachelectomy can be proposed for pregnant women diagnosed with FIGO IA2-IB tumors < 2 cm with negative nodes [11,36] (Figure 2). The data from the SHAPE trial presented at the 2023 ASCO Annual Meeting may comfort the surgeon in de-escalating surgery towards a simple trachelectomy when the selection criteria are met (IA2-IB1 squamous cell/adenocarcinoma/adenosquamous carcinoma < 2 cm with less than 50% stromal invasion and negative nodes) [54].

One of the greatest motivations to perform radical trachelectomy (RT) during pregnancy is to enable both continuation of the pregnancy and cancer treatment without delay. From an oncological point of view, a systematic review totaling more than 600 cases confirmed an overall recurrence rate < 5% and a death rate < 3%, demonstrating the safety of this approach for small invasive cervical tumors [55]. RT in pregnancy has been investigated by many teams, as first-line therapy for patients diagnosed with tumors <2 cm (≤IB1 according to FIGO 2018) [33,56,57,58,59,60,61,62,63,64,65,66] or, in rarer situations, for patients diagnosed with tumors >2 cm (≥IB2 according to FIGO 2018) [67,68,69,70,71,72,73,74,75]. RT can be performed either through abdominal [58,59,60,63,66,67,69,72,74,76] or vaginal approach (Dargent’s method) [56,57,61,71,73,75,77,78], each technique being associated with its own advantages and drawbacks. Vaginal approach allows for less manipulation of the pregnant uterus and thus may carry a lower risk of spontaneous abortion. Abdominal approach allows for a wider resection of the parametrium (including contiguous parametrial nodes), up to the level of type III hysterectomy [79]. Some teams believe that large parametrial resection may not be mandatory in this setting [57], but a higher recurrence rate and cancer-related death were demonstrated with vaginal approach [80]. Whatever the approach, trachelectomy remains a heavy surgery associated with long operating times ranging from 3.5 [59] to 7.5 h [74] and blood loss of up to 2.5 L [72]. Fetal risks for RT performed during pregnancy include spontaneous abortion, premature rupture of the membranes, preterm delivery, chorioamniotitis, and fetal death. Many teams attempted to adapt their procedure when performed during pregnancy. Iwami et al. proposed to perform a daily vaginal disinfection with povidone iodine, bed rest, and administration of ritodrine (β2 adrenoreceptor agonist) and ulinastatin (urinary trypsin inhibitor) vaginal suppository until delivery, and report improved pregnancy courses with this strategy [57]. The administration of vaginal progesterone did not reduce the rate of preterm birth in a case–control study led by Sato et al. [81]. Abdominal radical trachelectomy with ligation of both uterine arteries has been suggested to be a risk for premature birth, intrauterine growth retardation (IUGR), and death, due to increased fetal hypoxia [67]. Some teams advocates in favor of either bilateral [66,72] or unilateral [59,74,76] preservation. Recent data, with procedures adapted to pregnancy, seem to support the safety for mother and child of both vaginal and abdominal approaches, but the small number of patients hampers these conclusions. Thus, radical trachelectomy performed through either vaginal or abdominal approach is still not recommended during pregnancy due to poor fetal outcomes, with a high risk of early abortions and neural tube defects when performed during the first trimester of pregnancy [11].

##### Cesarean-Radical Hysterectomy (CRH) vs. Delayed Radical Hysterectomy (RH)

CRH was the preferred modality of surgical therapy across studies [56,59,68,82,83,84,85,86,87,88,89,90,91,92,93,94,95,96,97,98,99,100,101,102,103,104,105,106,107,108,109,110,111,112,113,114]. Its main indications were residual disease after radical trachelectomy, IB1 tumors in patients who do not wish to undergo fertility-preservation surgery, or for IB2-3 tumors after neoadjuvant chemotherapy (Figure 2). Allowing performance of both delivery and cancer treatment in the same operating time avoids a further delay, which could be deleterious in terms of cancer prognosis. Excellent oncological outcomes were demonstrated across trials, with only 14 recurrences out of 189 patients [86,106,107,110,115,116]. No survival difference was found between CRH compared to RH performed among non-pregnant women [99,110]. CRH is not necessary in situations when an in sano resection with either conization or trachelectomy has already been performed.

Compared to delayed RH, CRH is at risk of significantly higher blood loss (2033 cc vs. 425 cc) [99]. In a national survey, women undergoing CRH had a higher rate of perioperative complications compared to those undergoing RH (45.1% vs. 32.1%), consisting mostly in hemorrhage, bowel obstruction, and pyelonephritis. Mortality rates were similar between the two groups (0% vs. 0.2%) [117]. To date, ESMO guidelines suggest to proceed with radical surgery concomitantly to cesarean delivery in qualified centers [118].

##### Ovarian Transposition

Ovarian transposition has been performed at the time of the hysterectomy by a few teams in order to preserve hormonal production when adjuvant radiation therapy was deemed likely [84,87,94,98,119,120,121]. Ovarian metastasis from cervical cancer remains rare [122]. In a nationwide multicenter study examining consecutive cases of surgically treated women with clinical stage IB-IIB cervical cancer, onset of ovarian metastasis was associated with adenocarcinoma histology, lympho-vascular space invasion, uterine corpus tumor invasion, and nodal metastases. In the absence of these five risk factors, ovarian metastasis remained anecdotal (0.14%) [123]. An accurate selection of patients is necessary in this setting, and to date no specific guidelines have been made on this topic.

##### Pelvic Lymph Node Dissection (PNLD) vs. Sentinel Lymph Node Dissection (SLND)

Pathologic assessment of pelvic +/− para-aortic lymph nodes is mandatory before starting any treatment, as its results will guide therapeutic decisions for both the oncological approach and pregnancy management. The current guidelines on cervical cancer during pregnancy advocate its realization for patients diagnosed with IB1 or IB2 tumors before 22 weeks of gestation [12] (Figure 2). Usually, PLND is not performed after this term because the dimension of the gravid uterus may preclude the ability to access the pelvic lymph nodes. When performed by experienced surgeons, this technique does present few maternal or fetal morbidity or mortality risks. Only one case of spontaneous abortion occurred at 12 weeks of pregnancy, yet lymphadenectomy was performed in association with radical trachelectomy [71].

Few data are available with regards to SLND for pregnant cervical cancer patients, but this technique represents an attractive alternative to PLND, as it could enable a further decrease in intervention time and blood loss. Sentinel node mapping using radioactive tracers is contraindicated for cervical cancer—although it is not for vulvar cancer. Indocyanine green may be promising in this setting, as it has already been shown not to cross the placenta after intravenous injection [124]. Papadia et al. reported SNLD performed with indocyanine green on two patients and recorded no adverse events [125]. The scarcity of data on this subject precludes any conclusion on the conduction of sentinel lymph node dissection during pregnancy, and further trials are warranted before its introduction in routine care.

#### 4.1.3. Chemotherapy

Neoadjuvant chemotherapy (NAC) is the preferred option for patients diagnosed with FIGO 2018 IA2-IB1 cervical tumors after 22 weeks of gestation and for patients diagnosed with IB2-IB3 cervical tumors regardless of the term [12] (Figure 2). NAC allows for a double compromise: treating the cancer while postponing local treatment until fetal maturity.

Chemotherapy administered during pregnancy is associated with specific risks with regards to the pregnancy. Chemotherapy is contra-indicated during the first trimester of pregnancy, being associated with a 10 to 20% rate of malformations and high-risk of miscarriage [126]. After 14 weeks of gestation, its administration is feasible and associated with a low rate of fetal adverse effects. Based on the results of several prospective trials, Platinum-based chemotherapy with either Paclitaxel or Docetaxel represents the most prescribed regimen in the neo-adjuvant setting [127]. This regimen has been widely used across trials [62,69,82,83,84,85,87,88,90,92,103,104,106,108,109,111,112,121,128,129,130,131,132,133,134,135,136,137,138,139,140] and was usually associated with a favorable tolerance profile, with no report of grade 3–4 adverse events. General guidelines advocate in favor of the use of Carboplatin instead of Cisplatin in pregnant women to avoid ototoxicity in children exposed in utero. Taxanes during pregnancy are deemed safe, with their main adverse events consisting mostly of non-severe anaphylactic reactions and occurrence of preterm contractions [106,141].

Caution should also be exerted with regard to drugs usually prescribed during chemotherapy. Premedication with corticosteroids and Ondansetron appears to be safe regardless of the term of the pregnancy, but insufficient data are available with regards to the use of Aprepitant. Growth factors are contraindicated, thereby emphasizing the crucial need for hematological monitoring and dose adjustment. Maternal anemia is a multifactorial issue (due to dilution, tumor bleeding, and the hematological toxicity of chemotherapy), thus requiring early detection and adequate supplementation.

Trials investigating the role of NAC for cervical cancer revealed promising results, with response rates ranging from 66% to 85% and considerable rates of pathological complete responses [142]. The EORTC 5594 trial demonstrated similar survival outcomes with NAC followed by surgery compared to chemoradiotherapy (CRT), with 5-year overall survival (OS) of 72% and 76% respectively. However, a significant proportion of patients could not undergo the planned surgery (24%), due to either toxicity, progression, or an insufficient response to NAC [143]. NAC followed by surgery also demonstrated inferior disease-free survival compared to CRT in randomized trials, with 18.6% experiencing local first recurrence in the NAC group vs. 13.5% in the CRT group [144]. Additionally, a report of rapid progression on NAC in a pregnant patient has been published in a patient with locally advanced disease [145].

Caution needs to be exerted when NAC is advocated as first-line therapy for pregnant cervical cancer patients, given the poor prognosis in the event of tumor progression. Patients diagnosed with bulky tumors may be more appropriately counselled towards CRT associated with termination of pregnancy, especially if the diagnosis is made during the first trimester (Figure 2). If the patient wishes to continue the pregnancy, both clinical and MRI assessment must be arranged at each cycle to eliminate tumor progression.

#### 4.1.4. Radiation Therapy

Pelvic radiation therapy is contraindicated during pregnancy as it is known to induce fetal death and spontaneous abortion after 2–3 weeks of treatment [146]. CRT has been performed for locally advanced tumors after premature delivery in patients diagnosed during their third trimester of pregnancy [119,147]. Although current guidelines recommend termination of pregnancy for locally advanced tumors, some patients choose to proceed with the pregnancy. In this situation, some authors reported an approach using NAC until fetal maturity, followed by premature delivery and CRT [128,131,133,135,138] (Figure 2).

One team reported chemoradiation therapy (CRT) with fetuses in utero for advanced-stage cervical carcinoma diagnosed during the first trimester of pregnancy [145]. The patients were diagnosed with large tumors (>8 cm), which made uterine evacuation impossible through vaginal approach. After 3 weeks of CRT, tumor regression allowed vaginal evacuation, avoiding the risk of tumor spread with hysterotomy. CRT with the fetus in utero seems feasible without major short-term adverse events, either urinary or digestive. While this approach is not exempted from ethical dilemmas and psychological distress, it must be restricted to complex situations where the cancer spread requires immediate treatment.

#### 4.1.5. Delaying Definitive Treatment

Delaying definitive treatment to improve fetal outcomes, although beneficial to the fetus, may carry an additional risk of tumor progression. However, the clinical data are reassuring and do not report either significant disease progression or loss of chance during pregnancy. Morice et al. retrieved published data on delayed treatment and described the outcomes for 76 patients diagnosed mostly with localized tumors (96% rate of FIGO 2009 IB tumors). Even with a median delay of 16 weeks, excellent oncological outcomes were demonstrated, with a 95% survival rate at a mean follow-up of 37.5 months [148]. Amongst them, Lee et al. reported outcomes after delayed treatment for 12 patients. Two patients, diagnosed with FIGO IB2 and IB3 (FIGO 2018) cervical cancer, died of the disease, with a mean delays of treatment of 6 and 4 weeks, respectively [110]. Favero et al. reported the outcomes of 14 node-negative patients after an average treatment delay of 17 weeks. Nine of them underwent CRH, while five underwent RT approximately 6 weeks after delivery. All patients remained disease-free at a mean follow-up of 38 months [114]. Ishioka et al. reported planned delay of treatment for patients diagnosed with IB1-IIB cervical cancer. No apparent tumor growth was reported, although surgery had to be preempted for one patient due to pain (parametrial invasion), emphasizing the importance of patient selection in this setting [101].

According to the most recent guidelines, a delay in definitive treatment may be considered feasible for patients diagnosed with IA2-IB3 tumors diagnosed after 22 W, subject to close monitoring [12], with the aim to obtain fetal maturity (>37 weeks of gestation, WG) (Figure 2). The ESMO guidelines also support delayed treatment and careful monitoring of the patient at all early stages [118]. When progressive disease is observed, either termination of pregnancy or NACT should be advocated.

#### 4.1.6. Rare Histological Types

Few conclusions can be drawn from case reports relating to cervical cancer of rare histology diagnosed during pregnancy. In decreasing frequency order, we retrieved cases about treating neuroendocrine carcinomas [97,113,120,121,136,137,138,139,140,149,150,151,152,153,154], clear cell carcinomas [134,135,155], and sarcoma [98].

Most patients diagnosed with neuroendocrine tumors at the first or early second trimester of pregnancy underwent termination of pregnancy [121,136,151,154] to prevent any delay in cancer management. Three out of fifteen patients were diagnosed with stage FIGO 2018 IVB disease and died within months after the diagnosis [137,150,152]. Three additional patients diagnosed with localized disease died from either lung or liver metastases in less than a year [113,137,140], demonstrating the particularly poor prognosis of this disease.

Specific guidelines were published for the management of small cell cervical cancer in 2011. A multimodal approach is recommended for early stages, including surgical resection, pelvic radiotherapy, and chemotherapy [156]. Of the cases collected in Supplementary Table S1, most patients were diagnosed with locally advanced disease due to either vaginal or parametrial invasion and underwent NAC followed by radiation therapy [138,149,153,154]. All patients remained disease-free after a follow-up ranging from 14 months to 5 years. NAC consisted mostly of platinum-based regimens (with Etoposide/Paclitaxel) or poly-chemotherapy with Cyclophosphamide and Adriamycin. For metastatic disease, platinum-based chemotherapy was usually advocated. Even if initial response rates are high (50–70% in the non-pregnant population), recurrence and progression are frequent and the outcomes remained poor [156].

### 4.2. Obstetrical Management

#### Planned Delivery

Current guidelines advocate conducting the pregnancy to its full-term (>37 WG). A 3-week interval is usually advocated between the last chemotherapy and delivery to avoid hematopoietic suppression. A full blood count is mandatory in order to evaluate the degree of anemia, thrombopenia, and neutropenia—all the more if delivery and cancer treatment are planned at the same time.

Among the 27 patients undergoing vaginal delivery [68,86,94,99,107,113,115,140,150,157,158,159,160,161,162,163,164,165], 9 patients experienced recurrence at the episiotomy site [86,115,157,158,162,163,164,165]. In other situations, cervical cancer was revealed by metastasis at the episiotomy site several months after delivery, having remained occult during the pregnancy [157,162,164,166,167,168,169,170]. The metastatic lesion was described as either a swelling, a nodular or ulcerative lesion, or even an abscess. The etiology of these recurrences appears to be related to local seeding at the time of delivery, rather than metastasis through a vascular or lymphatic route. Vaginal delivery was the most significant predictor of recurrence in a large cohort of 83 pregnant women, with an odds ratio (OR) of 6.9. On the other hand, only one report described a recurrence where Cesarean section had been performed [171]. Cesarean delivery should be the preferred method in pregnant women diagnosed with invasive cervical cancer [12].

In a large series of women in California with pregnancy-associated cervical cancer, higher rates of cesarean section (OR 3.7; 95% CI 2.6, 5.2), hospitalization > 5 days (maternal: OR 14.1; 95% CI 9.2, 21.5 neonatal: OR 5.2; 95% CI 3.6, 7.5), low birth weight (OR 5.5; 95% CI 3.7, 8.1), very low birth weight (OR 6.9; 95% CI 3.7, 12.8), prematurity (OR 4.7; 95% CI 3.2, 6.7), and fetal deaths (OR 5.5; 95% CI 2.0, 14.8) were reported compared to non-cancer pregnant controls [172].

### 4.3. Pediatric Management

#### 4.3.1. In Utero Surveillance

At the time of cancer diagnosis, it is necessary to assess the exact term of the pregnancy and to exclude fetal anomalies through ultrasonography and specific screening, according to guidelines. During cancer treatment, specific attention should be given to fetal anemia after chemotherapy administration, as its complications range from fetal hydrops to fetal death. Currently, peak systolic velocimetry of the middle cerebral artery is considered as the best method and brings the advantage of being reproducible throughout the pregnancy [173].

#### 4.3.2. Pediatric Management

Most of the concerns for the neonates are related to premature delivery. Morbidity attributable to prematurity includes respiratory distress syndromes, ranging from transient oxygen need to invasive ventilation [58,75,82,89,109,129,135,137,139] and including hypoglycemia [135,139] and necrotizing enterocolitis [75]. No death due to premature delivery was reported in the setting of cervical cancer associated with pregnancy. During the past decades, neonatal intensive care has been improved significantly and both infant morbidity and mortality have continued to decline, with survival rates above 90% for children born after their 27th week of gestation in a recent national survey [174]. Still, the ESMO recommends delivery at term (>37 weeks) in order to decrease morbidity, which can already be significant due to cancer treatment [127]. Specialized care (neonatology—intensive care) is advised after delivery and needs to be tailored to each clinical course.

Although extremely rare, transmission of cervical cancer from mother to child has been reported in the literature [152,175]. Two routes of mother-to-child cancer metastases can be identified. Hematological dissemination can occur through placental metastases and has been described for aggressive cancers such as melanoma and small cell cervical carcinomas [152]. Recently, two cases of lung metastases from cervical cancer have been reported, raising concerns about the possibility of metastasis after inhalation of cancer cells at the time of delivery [175]. In these specific situations, a follow-up with a pediatrician from a reference center should be initiated, along with regular clinical examination. Atypical and persistent symptoms in a child with this type of history require the utmost vigilance and targeted examinations.

Precautions should be taken with regards to breastfeeding in patients who have received chemotherapy during pregnancy. Cisplatin has been detected in breast milk at concentrations up to 10% of those in the maternal blood [109]. Breastfeeding is not contraindicated after cancer treatment, provided that there is a minimum delay of 3 weeks between the last chemotherapy and its beginning [12].

Due to their low molecular weight (less than 600 kDa), most cytotoxic agents cross the placenta [176]. Cisplatin concentrations in the umbilical cord and amniotic fluid were shown to be up to 31–65% and 13–42% of the amount in the maternal blood, respectively [112,177]. The paclitaxel concentration in the fetal umbilical cord has been shown to rise up to 15% of the maternal blood concentration 3 h after paclitaxel administration. Even if very few toxicities have been reported for both fetuses and children exposed to chemotherapeutic agents during pregnancy, most studies are hampered by their short follow-up and do not report on the long-term potential complications. After in utero chemotherapy exposure, both neonates and infants need specific examination performed by a trained neonatologist or pediatrician. Hematological, liver, and renal parameters should be checked. In case of Cisplatin exposure, special attention must be paid with respect to hearing function and cardiac malformations [178]. Induced malignancies in the child, such as rhabdomyosarcoma or acute myeloid leukemia, have been reported in the literature after in utero exposure to platinum and paclitaxel [85,139]. While these events remain anecdotal, their severity requires them to be kept in mind.

### 4.4. Prognosis

Several factors may be associated with an impaired prognosis for cancer in pregnant women. First, diagnostic difficulties may arise due to the limitations of certain procedures during pregnancy (PET/CT for instance) that may ultimately result in under-staging. However, surgical staging via PNLD, the gold-standard method for lymph node evaluation, can be performed until the end of the second trimester of pregnancy. Second, the contraindication to perform local procedures (radiotherapy, radical hysterectomy) may worsen the prognosis if the alternative approach (either delay or NAC) is inappropriate. Nonetheless, the oncological outcomes of cervical cancer diagnosed during pregnancy seem relatively similar with those of cervical cancer diagnosed in the global population. Large epidemiological studies showed similar maternal and neonatal morbidity for patients diagnosed during pregnancy than for non-pregnant patients [15,179,180,181,182,183]. Only one trial showed a 1.24 age-adjusted hazards ratio for cancer-related deaths, compared with non-pregnant patients (confidence interval between 0.84 and 1.81) [184]. The limitations of these national surveys include their retrospective nature and their long inclusion period. Standardization of the management of patients with cervical cancer during pregnancy through the development of international guidelines will likely improve the management of these patients.

## 5. Discussion

The management of cervical cancer or pre-cancerous lesions during pregnancy is complex and can jeopardize both the pregnancy and maternal prognosis. Additionally, it seems that the very presence of HPV lesions favors obstetrical complications, even if the precise physiopathology is not well known [185,186]. It is therefore essential to act on primary prevention. The real-life efficacies of HPV vaccination programs have been demonstrated in several national surveys, with significant reductions in the risk of invasive cervical cancer [187,188]. While health institutions in France recently opted in favor of an enlargement of the population targeted for HPV vaccination, some authors even propose a vaccination program during the postpartum period [189]. The second prevention lever against HPV lesions is represented by their early detection by cervical smear. Since 2019, HPV testing has replaced the classic cytology from 30 to 65 years in screening in France [190]. While the classic obstacles to this screening are the fear of the gynecological examination and the absence of gynecological follow-up, urine HPV self-testing could replace the classic smear test for those populations that escape screening and has already been validated in pregnant women [191].

Advances in cervical cancer in general may also benefit patients diagnosed during pregnancy. Recent data on the safety of MRI at all terms (without Gadolinium injection) and laparoscopic lymph-node staging (up to a certain term and in trained teams) have made it possible to improve staging and to more easily offer active surveillance.

Locally advanced cervical cancer during pregnancy is one of the most challenging situations, as it theoretically requires CRT, which is incompatible with preservation of the pregnancy. For women who wish to proceed with the pregnancy, NAC represents the only therapeutic option, but there is a risk of tumor progression and altered maternal prognosis. One of the options could be to potentiate the tumor response with an additional chemotherapeutic agent to maintain the pregnancy more serenely. One of the leads could be the use of Bevacizumab, which has demonstrated its value in PFS and OS in metastatic cervical cancer [192]. However, IV antiangiogenic drugs have not been the subject of any publications during pregnancy and could potentially be very toxic for both mother and fetus (vascular, renal, pre-eclampsia). Long-standing experience with thalidomide suggests that great caution should be exercised, and antiangiogenic drugs are therefore contraindicated during pregnancy. PDL1 inhibitors have recently shown efficacy in PFS and OS in first-line metastatic cervical cancer [193]. They are also of growing interest as adjuvant therapy after CRT in locally advanced cancers [194,195,196,197]. In these challenging situations, one could imagine proposing immunotherapy as a complement to NAC to increase the response rate in pregnant women. However, as treatment with anti-PD-1 and anti-PD-L1 monoclonal antibodies enhance the functional activity of T cells towards tumor cells, they simultaneously abrogate the established fetomaternal immunotolerance [198]. Preclinical data show that anti-PD1 and PDL-1 cross the placental barrier, especially in the 2nd and 3rd trimesters. Additionally, adverse effects on both the fetus and pregnancy have been described in animals and in some case reports [198]. Thus, preliminary data on checkpoint inhibitors and pregnancy are scarce at best and even worrisome, discouraging their further implementation in clinical trials. As Tisotumab–Vedotin recently showed a durable antitumor response in recurrent or metastatic cervical cancer in a phase II trial [199] and was recently granted accelerated approval by the FDA, further safety data on pregnant women will likely be provided in the coming years. Cervical cancer during pregnancy requires management in expert centers to optimize maternal oncological treatment while ensuring fetal development. Based on our experience, we aimed to summarize our “red flags” for both mother and child in order to fill the gap with the current guidelines (Table 2).

A multidisciplinary team involving a trained gynecologist, oncologist, radiation oncologist, neonatologist, pediatrician, and psychologist is required at all stages of the treatment. In France, the “Cancer Associé à La Grossesse” (CALG) network provides specific counseling and a dedicated tumor board for the management of women diagnosed with cancer during pregnancy.

Our systematic review carries some limitations. Mostly, it is based on either case reports or small series, with no prospective data existing on this topic. Also, the search modality intended to be quite complete, but the use of the keyword “cervix”, with no additional search, might result in the loss of some information—despite a thorough search within the bibliography of the selected articles.

## 6. Conclusions

Cervical cancers occurring during pregnancy require a multidisciplinary team and management in expert centers. Therapeutic advances in the past decades have made it possible to improve both fetus and infant prognosis through the reduction of the termination of pregnancy rate and improvement of neonatology techniques. The occurrence of cervical cancer during pregnancy does not appear to adversely affect maternal prognosis, whether because of increased aggressiveness or inadequate treatment. More investigations are necessary with regards to imaging modalities such as PET-MRI and surgical techniques such a RT or SLND, since they represent promising alternatives. Long-term follow-up of infants, especially those exposed to cytotoxic agents in utero, is necessary.

## Figures and Tables

**Table 1:** Fetal risk caused by ionizing radiation, according to the dose and term of pregnancy.

	0–100 mGy	100–500 mGy	>500 mGy
0–2 WG	Death of the embryo		
2–8 WG	No demonstrated effects	Risk of malformation	
8–24 WG	No demonstrated effects	Risk of reducing intellectual abilities	High risk of mental retardation
24–39 WG	No demonstrated effects	Decrease of all determinist side effects	

Abbreviations: mGy = miliGray, WG = week of gestation.

**Table 2:** “Red flags” for diagnosis and follow-up of cervical cancer diagnosed during pregnancy.

**At the time of diagnosis:** *General care* Evaluate maternal psychological distress—refer for psychological support *Tumor staging* - Tumor staging (clinical evaluation, MRI without gadolinium) - Evaluate the need for surgical lymph node dissection *Obstetrical care* - Evaluate the term of the pregnancy (ultrasonography) - Evaluate fetal pre-existing conditions—prenatal screening and diagnosis (ultrasonography, blood samples, amniocentesis if necessary)
**Treatment during pregnancy:** *General care* Evaluate maternal psychological distress—refer for psychological support *Chemotherapy* - Exclude tumor progression under neoadjuvant chemotherapy (MRI without gadolinium) *Obstetrical care* - Evaluate fetal growth (ultrasonography) - Exclude fetal malformation (ultrasonography) - Exclude fetal anemia (doppler of the middle cerebral artery)
**Before delivery:** *General care* - Ensure an interval of 3 weeks between chemotherapy and delivery—research maternal thrombopenia or neutropenia (blood samples) - Evaluate maternal psychological distress—refer for psychological support *Obstetrical care* - Plan delivery with neonatologist unit
**After delivery:** *General care* Evaluate maternal psychological distress—refer for psychological support *Tumor staging* - Tumor staging on both local, nodal, and metastatic level (MRI, PET/CT) - Evaluate the need for adjuvant therapy *Child care* Long-term follow-up required with a trained pediatrician, particularly if neoadjuvant chemotherapy was performed during pregnancy

Abbreviations: MRI = Magnetic Resonance Imaging, PET/CT = Positron Emission Tomography/Computed Tomography.

## Data Availability

Data are contained within the article and Supplementary Materials.

## Supplementary Materials

The following supporting information can be downloaded at: https://www.mdpi.com/article/10.3390/cancers16071341/s1, Table S1: case reports of cervical cancer diagnosed during pregnancy. Stage has been updated according to the FIGO 2018 classification; Table S2: series of cervical cancer diagnosed during pregnancy. Stage has been updated according to the FIGO 2018 classification.
