# Supplementary material for: Cervical Cancer Associated with Pregnancy: Current Challenges and Future Strategies
Source: Cancers (Basel). 2024 Mar 29;16(7):1341. doi: 10.3390/cancers16071341 (PMC11011172; doi:10.3390/cancers16071341)
Supplement: Supplementary file 1 [file cancers-16-01341-s001.zip › cancers-2901372-Table S2.pdf]

Supplementary Table S2 : series of cervical cancer diagnosed during pregnancy. Stage has been updated according to the FIGO 2018 classification

**Author**	**Number of patients**	**Histology**	**Stage**	**Trimester**	**Treatment**	**Delivery**	**Maternal outcome**	**Fetus outcomes**
Alouini et al., 2008	8	SCC (5)<br>ADK (3)	IB1 – IIIC1	1T – 3T<br>12W – 32W	RPL + PL first, as staging modality<br>Curative treatment depending on the stage of the disease afterwards	Cesarean	Died of disease for IIIC patients<br>Disease-free for stade I-II patients	1 miscarriage<br>7 healthy infants
Baltzer et al., 1990	40	NS	IB	12W (10)<br>22W (10)<br>36W (4)<br>Postpartum (16)	RH + PL<br>Radiotherapy if necessary	Cesarean	Better prognosis for patients diagnosed during pregnancy than post-partum	NS
Benhaim et al., 2007	2	SCC (2)	IIB<br>IVA	1T	ARCC with fetus in utero<br>Uterine curettage<br>BT<br>+/- surgery	Termination of pregnancy	Died of disease at 20 months – disease-free at 29 months	Termination of pregnancy
Bigelow et al., 2016	Case-control<br>28 pregnant<br>52 non-pregnant	SCC / ADK	IB1 (56%)<br>II (14%)<br>III-IV (11%)	Average : 2T / 17W	RH after delivery or few weeks after	Termination of pregnancy / miscarriage (1T) (7)<br>Mean delivery : 36W<br>Vaginal delivery (4)	No difference in survival between groups	25% termination of pregnancy<br>No significant fetal growth restriction for other babies<br>Healthy infants

Carillon et al., 2011	5	SCC (3)<br>ADK (1)<br>SmCC (1)	IB1 (80%)	1T (1)<br>2T (3)<br>3T (1)	NAC (3)<br>ARCC (4)<br>Palliative chemotherapy (1)	Median cesarean at 32W	1 died of disease at 12 months (neuroendocrine carcinoma)	2 respiratory distress<br>Healthy infants
El Mazghi et al., 2014	5	Poorly differentiated SCC (5)	IB1 (3)<br>IB3 (2)	1T (1)<br>2T (1)<br>3T (3)	ARCC (2)<br>RH + PL + Radiotherapy (3)	Termination of pregnancy (2)<br>34-37W : Cesarean (3)	Disease-free at median 36 months	NS
Favero et al., 2010	18	SCC (9)<br>ADK (9)	IA1 (2)<br>1A2 (1)<br>1B1 (13)<br>1B2 (1)<br>2A (1)	1T (4)<br>2T (14)	PL first, as staging modality	Cesarean (14)	Disease-free at median 38 months	Healthy infants (14)
Ferriaoli et al., 2012	5	SCC (3)<br>ADK (2)	IA2 (1)<br>IB1 (3)<br>IIIC2 (1)	1T (3)<br>2T (2)	RT + PL (5)<br>Adjuvant RH + ARCC (1)	Termination of pregnancy (1)<br>Spontaneous abortion (2)<br>Cesarean (3)	Died of disease at 3 years (1)<br>Disease-free at median 191 months (4)	Healthy infants (3)
Fruscio et al., 2012	9	SCC (7)<br>ADK (2)<br>Poorly differentiated (7)	IB2 (4)<br>IB3 (5)	1T (1)<br>2T (8)	NAC after 16 weeks of gestation (Cisplatin / cisplatin + Navelbine) (9)<br>After delivery : RH (9)<br>Adjuvant RT (1)<br>Adjuvant ARCC + BT (1)	30-36W : Cesarean (9)	Died of disease at 27 months (2)<br>Local relapse (2)<br>Disease-free after pregnancy (5/9)	Healthy infants (9)
Germann et al., 2005	21	SCC (20)<br>ADK (1)	IB1 (14)<br>IB2 (1)<br>IIB (5)<br>IVA (1)<br><br>After lymph node	1T (13)<br>2T (5)<br>3T (2)<br>Post-partum (1)	RH + PL + BT (10)<br>RH + PL + RT + BT (5)<br>RH + PL + RT + BT + AC (3)<br>Radiotherapy + RH + PL + BT (1)	Termination of pregnancy (9)<br>Spontaneous abortion (1)<br>Vaginal delivery (3)	Disease-free (14)<br>Died of disease (4)<br>Lost of follow-up (2)	Healthy infants (10)

			evaluation :<br>IIIC1 (7)<br>IIIC2 (2)		Radiotherapy + RH + PL + RT + BR + AC (1)<br>arc + BT (1)			
Huang et al., 2021	4	SCC (4)<br>ADK (1)	IB2 (3)<br>IB3 (1)<br><br>After lymph node evaluation<br>IIIC1 (2)	2T (1)<br>3T (1)	NAC (Cisplatin + Paclitaxel) (4)<br>After delivery : RH + PL (4)<br>Rdiotherapy (4)	Cesarean (4)	Disease-free at 10-56 months (4)	Healthy infant (4)
Ishioka et al., 2009	7	SCC (6)<br>ADK (1)	IB1 (2)<br>IB2 (3)<br>IB3 (1)<br>NS (1)	1T (3)<br>2T (2)<br>3T (2)	RH + PL with fetus in situ (2)<br>NAC (1)<br>RH + PL after delivery (5)<br>AC (3)	Termination of pregnancy (2)<br>Cesarean (5)	NS	Healthy infants (5)
Lanowska et al., 2011	8	SCC (5)<br>ADK (3)	IB1 (7)<br>IVB (1)	2T (8)	PL (8)<br>NAC (Cisplatin) (8)<br>After delivery : RH (8)	Csarean (8)	Disease-free (8)	Transient respiratory distress (7)<br>Healthy infants (9) (twins)
Lee et al., 2008	40	SCC (30)<br>ADK (6)<br>Adenosquamous (1)<br>Others (3)	IA1 (6)<br>IB1 (16)<br>IB2 (9)<br>IIA (4)<br>IIB (3)<br>IIIB (1)<br>IVA (1)	1T (18)<br>2T (14)<br>3T (7)	RH + PL with fetus in situ (17)<br>RH + PL after delivery (7)<br>ARCC with fetus in situ (2)<br>RAT after delivery (1)<br>Conization after termination of pregnancy (1)	NS	Died of disease (2)<br>Disease-free (38)	Healthy infants (23)

					Delay of treatment (12)			
Li et al., 2011	2	SCC (2)	IB3 (2)	3T (2)	NAC (Cisplatin + Paclitaxel) (2)<br>After delivery : RH + PL (2)<br>ARCC (2)	Cesarean (2)	Disease-free at 13-21 months (2)	Healthy infant (2)
Martnitz et al., 2010	7	SCC	IB (6)<br>IIIC (1)	2T (7)	NAC (Cisplatin)<br>After delivery : RH + PL	Cesarean (7)	Disease-free at median follow-up of 7 months (7)	Healthy infants (8) (2 twins)
Papadia et al., 2015	2	Adenocarcinoma (1)<br>Adenosquamous (1)	IB2 (1)<br>IB1 (1)	2T (2)	SLN + PL (2)<br>Conization (1)<br>RAT+ AC (Carboplatin) + Radiotherapy (1)	Cesarean	Disease-free at 24 months (1)	Healthy infants (2)
Puchar et al., 2020	20	SCC (11)<br>ADK (8)<br>Undifferentiated (1)	IA (2)<br>IB (12)<br>II (2)<br>III (1)<br>Missing (3)	Median 23W (2T)	NAC (5)<br>RH (9)<br>Conization (5)<br>RAT (2)<br>No surgery (2)<br>Missing (2)<br>ARCC (8)<br>BT (2)	Termination of pregnancy (2)	NS	Healthy infants (18)
Ribeiro et al., 2012	3	SCC (2)<br>ADK (1)	IB2 (3)	2T (3)	RH + PLwith fetus in situ (1)<br>RH + PL after delivery (2)	Termination of pregnancy (1)<br>Cesarean (2)	Disease-free at 6 months - 5 years (2)<br>Vaginal recurrence (1)	Healthy infant (2)
Ricci et al., 2016	4	SCC (4)	IB2 (2)<br>IIA (1)<br>IIB (1)	1T (1)<br>2T (3)	NAC (Cisplatin / Carboplatin + Paclitaxel) (4)<br>After delivery : RH + PL (4)<br>ARCC (2) + BT (1)	Cesarean (4)	Died of disease at 2 years (1)<br>Disease-free (3)	Healthy infants (4)

					RT (1)			
Rodolakis et al., 2018 (serie and review)	26	ADK (7)<br>Adenosquamous (1)<br>SCC (18)	IA2 (2)<br>IB1 (22)<br>IB2 (2)	1T (8)<br>2T (18)	RVT + PL	Miscarriage (5: 4 during 1T, 1 during 2T)<br>Cesarean (21)	Died of disease (2)<br>Disease-free (24)	Death of the infant (1)<br>Healthy infants (20)
Salvo et al., 2018	5	ADK(3)<br>SCC (2)	IB2 (5)	1T (1)<br>2T (4)	Simple trachelectomy + PL (5)<br>AC (1)	Cesarean (5)	Disease-free at 18-168 months	Healthy infants
Takushi et al., 2002	28	SCC (27)<br>ADK (1)	IA1 (9)<br>IA2 (3)<br>IB1 (6)<br>IB2 (4)<br>IIA (1)<br>IIB (3)<br>IIIB (2)	<22W (20)<br>22-36W (4)<br>>36W (1)<br>Post-partum (3)	RH after termination of pregnancy (3)<br>RH with fetus in situ (8)<br>After delivery : RH (1)<br>After delivery : RT (1)<br>Delay of treatment after delivery (12)	NS	Disease-free (15)	Healthy infants (NNS)
Umemoto et al., 2019	5	ADK (3)<br>SCC (2)	IB1 (4)<br>IB2 (1)	2T (4)<br>3T (1)	RVT + PL (5)<br>After delivery : RH + AC (Carboplatin + Paclitaxel) (1)	Cesarean (5)	Local recurrence during pregnancy (1)<br>Disease-free (4)	NS
Ungar et al., 2006	5	SCC (5)	IB1 (5)	1T (4)<br>2T (1)	RAT + PL	Cesarean (2)<br>Miscarriage (3)	Disease-free at a median 40 months (5)	NS
Van Vliet et al., 1998	12	SCC (8)<br>ADK (4)	IB (7)<br>IIA (1)<br>IIB (1)<br>IIIC (3)	NS	Delay of treatment median 3.5 weeks (6)<br>RH with fetus in situ (3)<br>RH with fetus in situ + Radiotherapy (3)	Vaginal delivery (1)<br>Cesarean (5)	Died of disease (2)<br>Disease-free (10)	Healthy infants (6)

					After delivery :<br>RH (4)<br>Radiotherapy (2)			
Vercellino et al., 2013	32	SCC (17)<br>ADK (15)	IA1 (3)<br>IA2 (7)<br>IB1 (17)<br>IB2 (4)<br>IIA (1)<br><br>After staging :<br>IIIC1 (4)	1T (4)<br>2T (28)	PL first, as staging modality<br>NAC (14)<br>After delivery : RH (28)<br>ARCC (4)	Cesarean (27)<br>Termination of pregnancy (5)	Disease-free (23/23)	Healthy infants (29) (twins)
Xia et al., 2014	20	SCC (18)<br>Adenosquamous (1)<br>SmCC(1)	IS – IIA (16)<br>IIB-IIIB (4)	1T (8<br>2T (4)<br>3T (2)<br>Post-partum (6)	Surgery (15)<br>Radiotherapy/ ARCC (4)<br>Palliative chemotherapy (1)	Vaginal delivery (3)	Disease-free (14)<br>Died of disease (5)<br>Lost of follow-up (1)	NS
Yoshihara et al., 2018	6	SCC (6)	IB1 (6)	Median 15W	RT + PL(6)<br>After delivery : AC (Carboplatin + Paclitaxel) (4)	Cesarean (6)	Disease-free at 3-41months (6)	Healthy infant (6)
Zhang et al., 2015	20	SCC (18)<br>Adenosquamous (1)<br>SmCC (1)	IS (1)<br>1A1 (1)<br>IB1 (5)<br>IB2 (1)<br>IIA (8)<br>IIB (3)<br>IIIB (1)	1T (8)<br>2T (4)<br>3T (2)<br>Post-partum (6)	NAC (3)<br>Surgery (15)<br>RT/ARCC (5)	Vaginal delivery (3)<br>Cesarean (4)<br>Termination of pregnancy (13)	Died of disease (5)<br>Disease-free (15)	Retard of growth (1)<br>Healthy infants (6)

Abbreviations: SCC = Squamous Cell Carcinoma, RAT = Radical Abdominal Trachelectomy, PL = Pelvic Lymphadenectomy, TH = Radical Hysterectomy, T = trimester, W = weeks, NAC = Neo Adjuvant Chemotherapy, AC= Adjuvant Chemotherapy, SmCC = Small Cell Carcinoma, CCC = Clear Cell Carcinoma,

RT = Radiotherapy, BT = Brachytherapy, ARCC = , PNET = Primitive neuroectodermal tumor, RT =Radical Trachelectomy, RPL = Retroperitoneal Lymphadenectomy, SLN = Sentinel Lymph Node, LLETZ = Large loop excision of the transformation zone, RVT= Radical Vaginal Trachelectomy
