# Supplementary material for: Cervical Cancer Associated with Pregnancy: Current Challenges and Future Strategies
Source: Cancers (Basel). 2024 Mar 29;16(7):1341. doi: 10.3390/cancers16071341 (PMC11011172; doi:10.3390/cancers16071341)
Supplement: Supplementary file 1 [file cancers-16-01341-s001.zip › cancers-2901372-Talbe S1.pdf]

Supplementary Table S1: case reports of cervical cancer diagnosed during pregnancy. Stage has been updated according to the FIGO 2018 classification

Author	Histology	Stage	Trimester	Treatment	Delivery	Maternal outcome	Fetus outcomes
**Squamous Cell Carcinoma**							
Aoki et al., 2014	SCC	IB2	1T<br>12W	17W : RT + PL	38W : Csarean	Disease-free at 10 months	Healthy infant
Bader et al., 2007	Poorly differentiated SCC	IIIC1 (pelvic lymph nodes metastases)	2T<br>19W	23W : NAC (Cisplatin + Vincristine)<br>After delivery : RH + PL<br>After delivery: AC (Cisplatin + Vincristine + Bleomycin)	33W : Cesarean	Disease-free at 80 months	Respiratory distress syndrome at delivery<br>Healthy infant
Baloglu et al., 2007	Poorly differentiated SCC	IIIA (metastasis at the episiotomy site)	Post-partum	ARCC	Vaginal delivery with episiotomy	Disease-free	Healthy infant
Ben-Arie et al., 2004	Poorly differentiated SCC	IA2	2T<br>15W	17W : Repeat cervical conization + RPL<br>After delivery : RT	39W : Cesarean	Disease-free	Healthy infant
Bravo et al., 2012	SCC	IB2	1T	RT + PL	26W : Cesarean	Disease-free at 40 months	Healthy infant
Caluwaerts et al., 2006	Moderately differentiated SCC	IB1	2T<br>15W	17W : NAC (Cisplatin)<br>After delivery : RH + PL	32W : Cesarean	Disease-free at 6 months	Healthy infant
Can et al., 2013	SCC	28W : IIB<br>33W : IV (lung and placenta metastasis)	2T<br>28W	No treatment, lost to follow-up	34W : Vaginal delivery	Died of disease	Healthy infant

Dawood et al., 2013	SCC	IIB	Early 2T	NAC (Cisplatin)<br>After delivery :<br>ARCC + BT	28W : Cesarean	Died of disease at 18 months	Healthy infant
De vincenzo et al., 2018	Poorly differentiated SCC	IB2	3T<br>27W	NAC (Cisplatin + Paclitaxel)<br>After delivery : RH + PL<br>ARCC	35W : Cesarean	Disease-free	22 months : Acute myeloid leukemia
Dharan et al., 2009	SCC	IB1	2T<br>14W	22W : RT + PL	Termination of pregnancy	Disease-free	Termination of pregnancy
Enomoto et al., 2011	SCC	IIA	1T	RT + PL	37W : Cesarean	Disease-free at 6 months	Healthy infant
Fox et al., 2021	SCC	IIIC1 (pelvic lymph node)	3T<br>33W	After delivery :<br>PL + RPL + Ovarian transposition<br>ARCC<br>AC (Cisplatin + Paclitaxel + Bevacizumab)	34W : Cesarean	NS	Healthy infant
Goldman et al., 2003	Moderately differentiated SCC	IB2	3T<br>35W	After delivery : RH + PL + RPL	Vaginal delivery	Recurrence 5.5 years later at the episiotomy site	Healthy infant
Gurney et al., 2009	Well-differentiated SCC	IB1	2T<br>21W	After delivery : RAT + SLN	34W : Cesarean	Disease-free at 10 months	Healthy infant
Hoopmann et al., 2003	SCC	IB1	3T<br>38W	After delivery :<br>NAC (Carboplatin + Docetaxel)<br>RH + PL<br>AC (Carboplatin + Docetaxel)	Vaginal delivery	Recurrence after chemotherapy completion at the episiotomy scar	NS
Jadoul et al., 2011	SCC	IIIC2	3T<br>32W	Previous RAT<br>After delivery :<br>ARCC	33W : Cesarean	Died of disease	Healthy infant

Khalil et al., 1993	SCC	IIIB	After delivery	RT + BT	Vaginal delivery	Recurrence at the episiotomy site at 3 months<br>Died of disease	NS
Karam et al., 2006	Poorly differentiated SCC	IIB	2T<br>23W	NAC (Cisplatin)<br>After delivery : RH + PL + RPL + ovarian transposition<br>ARCC	33W : Cesarean	Disease-free at 14 months	Healthy infant
Karateke et al., 2010	SCC	IB3	2T<br>22W	2T : RAT<br>Then : RH	-	NS	Deceased during RT procedure
Kayahashi et al., 2018	Poorly differentiated SCC	IB3	2T<br>16W	NAC (Cisplatin + Paclitaxel) *complete response*<br>After delivery : RH + PL<br>ARCC	31W : Cesarean	Disease-free at 34 months	Respiratory distress syndrome<br>Generalized erythroderma (KID syndrome, non-imputable to chemotherapy)
Marana et al., 2001	SCC	IIB	2T<br>14W	NAC (Cisplatin + Bleomycin)<br>Lost of follow-up	38W : Cesarean	Died of disease at 1 year	Healthy infant
Palaia et al., 2007	Poorly differentiated SCC	IIB	2T<br>19W	NAC (Cisplatin + Paclitaxel)<br>After delivery : RH + PL	35W : Cesarean	Disease-free at 10 months	Healthy infant
Saso et al., 2015	SCC	IB1	2T<br>17W	19W : RT<br>After delivery : PL + RH	36W : Cesarean	Disease-free at 13 months	Healthy infant
Saunders et al., 1988	SCC	IV	2T<br>18W	Palliative RT (rib)	Termination of pregnancy	Died of disease at 6 weeks	Termination of pregnancy
Silva et al., 2005	Poorly differentiated SCC	IIIC1	2T<br>14W	After termination of pregnancy : lymphoscintigraphy<br>RH + PL<br>ARCC + BT	Termination of pregnancy	Disease-free at 30 months	Termination ofp regnancy

**Adenosquamous carcinoma**							
Hertel et al., 2001	Adenosquamous carcinoma	IIIC1 (pelvic lymph node)	2T<br>19W	2T : PL<br>2T : RH + RPL	Termination of pregnancy	NS	Healthy infant
Levy et al. 2020	Adenosquamous	IB3	2T<br>18W	NAC (Carboplatin + Paclitaxel)<br>After delivery : ARCC + BT	34W : Cesarean	NS	Healthy infant
Peculis et al., 2015	Poorly differentiated adenosquamous	IB3	2T<br>17W	20W : NAC (Cisplatin + Doxorubicin)<br>After delivery : RH + PL + ovarian transposition	34 : Cesarean	Disease-free at 20 months	Healthy infant
Schorge et al., 2017	Adenosquamous carcinoma	IB2	1T<br>9W	After delivery : RH + PL + bilateral oophorectomy<br>Radiotherapy	36W : Cesarean	Disease-free at 18 months	Respiratory distress<br>Healthy infant
**Adenocarcinoma**							
Dede et al., 2004	Villoglandular papillary adenocarcinoma	IB1	1T<br>8W	RH	Termination of pregnancy	Died of disease at 5 years	Termination of pregnancy
Favero et al., 2010	Moderately differentiated adenocarcinoma	IB1	1T<br>14W	17W : PL<br>20W : NAC (Cisplatin)	32W : Cesarean	Disease-free at 12 months	Healthy infants (twins)
Guo et al., 2020	Poorly differentiated adenocarcinoma	IB3	1T<br>13W	NAC (Carboplatin + Paclitaxel)<br>After delivery : RH + PL	35W : Cesarean	Disease-free at 4 months	Healthy infant
Heron et al., 2005	Villoglandular adenocarcinoma	IB1	3T<br>31W	After delivery : RH + PL + RPL	Vaginal delivery	Recurrence on the episiotomy scar at 4 years	Healthy infant
Hurteau et al., 1995	Villoglandular adenocarcinoma	IIB	2T<br>20W	After delivery : RH + PL + RPL	32W : Cesarean	Disease-free at 14 months	NS

Islam et al., 2012	Moderately differentiated adenocarcinoma	IB3	1T<br>10W	2T : NAC<br>After delivery :<br>RH + PL<br>ARCC	34W : Cesarean	Disease-free at 36 months	Healthy infant
Iwami et al., 2011	Adenocarcinoma	IB1	1T<br>11W	16W : PL + RT	34W : emergency cesarean (premature rupture of the membrane)	Disease-free at 14 months	Healthy infant
Kolomainen et al., 2013	Moderately differentiated adenocarcinoma	IB3	1T<br>2W	PL before pregnancy<br>16W : RT	25W : PROM	Disease-free at 46 months	Respiratory distress and necrotizing enterocolitis<br>Healthy infant
Kyrgiou et al., 2015	Moderately differentiated adenocarcinoma	IB1	1T<br>8W	9W : LLETZ<br>14W : RAT + PL	36W : Cesarean	Disease-free at 7 months	Temporary respiratory symptoms
Lavie et al., 2008	Well differentiated villoglandular adenocarcinoma	IB1	2T<br>13W	14W: conization<br>After delivery : RH + PL	37W : Cesarean	Disease-free at 18 months	Healthy infant
Lurie et al., 1991	Papillary serous adenocarcinoma	IB1	Immediately after delivery	After delivery<br>RH + PL + ovarian transposition	39W : spontaneous vaginal delivery	NS	Healthy infant
Marnitz et al., 2009	Adenocarcinoma	IB1	2T<br>14W	15W : PL<br>NAC (Cisplatin)<br>After delivery : RH	32W : Cesarean	32W : Cesarean	Healthy infants (twins)
Muallem et al., 2017	Adenocarcinoma	IB1	2T<br>25W	15W : Conization<br>25W : PL<br>After delivery : Total mesometrial resection	37W : Cesarean	Disease-free at 26 months	Healthy infant
Oliveira et al., 2018	Poorly differentiated adenocarcinoma	IIB	2T<br>20W	NAC (Cisplatin + Vincristine)<br>After delivery : RH + PL<br>BT	35W : Cesarean	NS	NS

Powell et al., 1999	Adenocarcinoma	IV (right ovary)	3T<br>34W	After delivery : right salpingo-oophorectomy<br>RH + PL + peritoneal biopsies + omentectomy + left sapingo-oophorectomy	34W : Cesarean	Disease-free at 1 year	Healthy infant
Salas et al. 2014	Poorly differentiated adenocarcinoma	IB3	2T<br>23W	23W : resection of the polypoid tumor<br>29W : RT<br>After delivery : RH + PL	34W : vaginal delivery	Disease-free at 22 months	Healthy infant
Sioutas et al., 2011	Differentiated ADK	IB1	1T	PL + RT	29W : Cesarean (chorioamniotitis)	Disease-free	Healthy infant
Van Calsteren et al., 2008	Adenocarcinoma	IB2	1T<br>12W	12W : Conization<br>15W : RPL	38W : vaginal delivery	Disease-free at 20 months	Healthy infant
Yi et al., 2013	Papillary mucinous adenocarcinoma	IB2	2T<br>18W	18W : PL + RAT<br>AC (Carboplatin + Paclitaxel)<br>After delivery : subtotal hysterectromy	34 W : Cesarean	Disease-free at 12 months	Healthy infant
**Other type of carcinoma**							
Abu-Rustum et al., 2010	Lymphoepithelioma-like carcinoma	IB1	2T<br>15W	15W : RAT + PL<br>39W : RH	39W : Cesarean	Disease-free at 12 months	Healthy infant
Mandic et al., 2009	Planocellular carcinoma	IB1	2T<br>17W	19W : RAT + PL	36W : Cesarean	Disease-free at 12 months	Healthy infant
Seamon et al., 2009	Glassy-cell carcinoma	IIIB	2T<br>23W	2T : NAC (Cisplatin + Vincristine)<br>After delivery : ARCC + BT<br>AC (Carboplatin + Paclitaxel)	31W : Cesarean	Disease-free at 4.1 year	Healthy infant
**Clear Cell Carcinoma**							

Ayhan et al., 2011	CCC	IB1	2T<br>18W	NAC : Cisplatin<br>32W : RH	32W : Cesarean	Disease-free at 36 months	Healthy infant (triplet pregnancy)
Boyd et al., 2009	CCC	IIB	2T<br>21W	25W :<br>NAC (Cisplatin)<br>After delivery :<br>Cisplatin<br>ARCC + BT	35W : Cesarean	Disease-free at 15 months	Oxygen need for 48H and hypoglycemia<br>Healthy infant
Terada et al., 2011	CCC	IIA	1T<br>10W	1T : RH + PL + bilateral oophorectomy	Termination of pregnancy	Disease-free	Termination of pregnancy
**Neuroendocrine Carcinoma**							
Balderston et al., 1998	SmCC	IIA	3T<br>30W	After delivery: NAC (Cisplatin + Etoposide / Vincristine + Dactinomycin + Cyclophosphamide)<br>Radiotherapy + BT<br>AC (Cisplatin + Etoposide)	30W : emergency cesarean	Disease-free at 5-years	Healthy infants (twins)
Canto et al., 2014	SmCC	IV	3T<br>36W	No treatment	36W : Vaginal delivery	Died of disease at 1 months post-partum	Healthy infant
Feng et al., 2021	PNET (poorly differentiated neuroblastoma)	IB1	2T<br>15W	21W : RH	Termination of pregnancy	Disease-free at 3-years	Termination of pregnancy
Herskovic et al., 2014	« neuroendocrine »	IV	Early 2T	NS	NS	Died of disease at 3 months	Metastatic neuroendocrine tumor<br>Died of disease
Komiyama et al., 2011	Atypical carcinoïd	IB3	Early 2T	13W : RH + PL<br>AC (Cisplatin + Paclitaxel + Etoposide)	Termination of pregnancy	Metastasis at 11 months<br>Died of disease at 19 months	Termination of pregnancy
Leung et al., 1999	SmCC	IIB	3T<br>31W	After delivery :<br>NAC (Cisplatin + Etoposide)	24W : Cesarean	Disease-free at 14 months	Healthy infant

				ARCC + BT<br>RH			
Li et al., 2009	Large cell neuroendocrine carcinoma	IIB	2T<br>18W	ARCC (with Cisplatin + Etoposide) + BT	Termination of pregnancy	Disease-freeat 21 months	Termination of pregnancy
Ohwada et al., 2001	SmCC	IB1	3T<br>27W	After delivery : RH + PL<br>AC (Cisplatin + Etoposide)	29W : Cesarean	Disease-free at 13 months	Healthy infant
Perrin et al., 1996	SmCC	IV	3T<br>25W<br>(misdiagnosed)	After delivery : excision of the mass<br>RH + PL + ovarian transposition	36W : Cesarean	NS	Healthy infant
Smyth et al.,2010	SmCC	IB3 bulky	2T<br>23W	2T : NAC (Adriamycin + Cyclophosphamide)<br>After delivery : NAC (Cisplatin + Etoposide)<br>ARCC	35W : Cesarean	NS	Mild growth retardation in utero<br>Healthy infant
Surbone et al., 2016	SmCC	IIB	2T<br>22W	28W : NAC (Cisplatin + Paclitaxel)<br>After delivery : RPL<br>6 months after : RH	34W : Cesarean	Disease-free at 6 years	Transient respiratory distress, hypoglycemia, hyperbilirubinaemia<br>At 5 years : retroperitoneal embryonal rhabdomyosarcoma
Tsao et al., 2001	PNET	IB3	1T<br>8W	NAC (Cyclophosphamide + Adriamycin + Vincristine / Ifosfamide + Etoposide)	Termination of pregnancy	NS	Termination of pregnancy

				RH + RPL + ovarian transposition<br>AC (Cyclophosphamide + Adriamycin + Vincristine / Ifosfamide + Etoposide)<br>Radiotherapy			
**Sarcoma**							
Meseci et al., 2013	Embryonal rhabdomyosarcoma	IB3	3T<br>30W	After delivery : RH + PL + ovarian transposition<br>AC (Vincristine + Actinomycine D)	35W : Cesarean	Disease-free at 45 months	NS

<u>Abbreviations:</u> SCC = Squamous Cell Carcinoma, RAT = Radical Abdominal Trachelectomy, PL = Pelvic Lymphadenectomy, TH = Radical Hysterectomy, T = trimester, W = weeks, NAC = Neo Adjuvant Chemotherapy, AC= Adjuvant Chemotherapy, SmCC = Small Cell Carcinoma, CCC = Clear Cell Carcinoma, RT = Radiotherapy, BT = Brachytherapy, ARCC = , PNET = Primitive neuroectodermal tumor, RT =Radical Trachelectomy, RPL = Retroperitoneal Lymphadenectomy, SLN = Sentinel Lymph Node, LLETZ = Large loop excision of the transformation zone
